# Interaction between glyphosate pesticide and amphiphilic peptides for colorimetric analysis†

**DOI:** 10.1039/d2na00345g

**Published:** 2022-07-28

**Authors:** Barbara B. Gerbelli, Pedro L. O. Filho, Bruna Cortez, Pedro T. Sodré, Mauricio D. Coutinho-Neto, Ian W. Hamley, Jani Seitsonen, Wendel A. Alves

**Affiliations:** University of Reading, Department of Chemistry Reading UK i.w.hamley@reading.ac.uk; Universidade Federal do ABC, Centro de Ciências Naturais e Humanas São Paulo SP Brazil wendel.alves@ufabc.edu.br; University of Copenhagen, Niels Bohr Institute Copenhagen Denmark; Universidade de São Paulo, Instituto de Física São Paulo SP Brazil; Nanomicroscopy Center, Aalto University Puumiehenkuja 2 FIN-02150 Espoo Finland

## Abstract

The large-scale use of glyphosate pesticides in food production has attracted attention due to environmental damage and toxicity risks. Several regulatory authorities have established safe limits or concentrations of these pesticides in water and various food products consumed daily. The irreversible inhibition of acetylcholinesterase (AChE) activity is one of the strategies used for pesticide detection. Herein, we found that lipopeptide sequences can act as biomimetic microenvironments of AChE, showing higher catalytic activities than natural enzymes in an aqueous solution, based on IC_50_ values. These biomolecules contain in the hydrophilic part the amino acids l-proline (P), l-arginine (R), l-tryptophan (W), and l-glycine (G), covalently linked to a hydrophobic part formed by one or two long aliphatic chains. The obtained materials are referred to as compounds 1 and 2, respectively. According to fluorescence assays, 2 is more hydrophobic than 1. The circular dichroism (CD) data present a significant difference in the molar ellipticity values, likely related to distinct conformations assumed by the proline residue in the lipopeptide supramolecular structure in solution. The morphological aspect was further characterized using small-angle X-ray scattering (SAXS) and cryogenic transmission electron microscopy (cryo-TEM), which showed that compounds 1 and 2 self-assembly into cylindrical and planar core–shell structures, respectively. The mimetic AchE behaviour of lipopeptides was confirmed by Ellman's hydrolysis reaction, where the proline residue in the peptides act as a nucleophilic scavenger of organophosphate pesticides. Moreover, the isothermal titration calorimetry (ITC) experiments revealed that host–guest interactions in both systems were dominated by enthalpically-driven thermodynamics. UV-vis kinetic experiments were performed to assess the inhibition of the lipopeptide catalytic activity and the IC_50_ values were obtained, and we found that the detection limit correlated with the increase in hydrophobicity of the lipopeptides, implying the micellization process is more favorable.

## Abstract 2

Self-assembled lipopeptides act function like the active site of acetylcholinesterase to detect organophosphorus pesticides.

## Graphical abstract

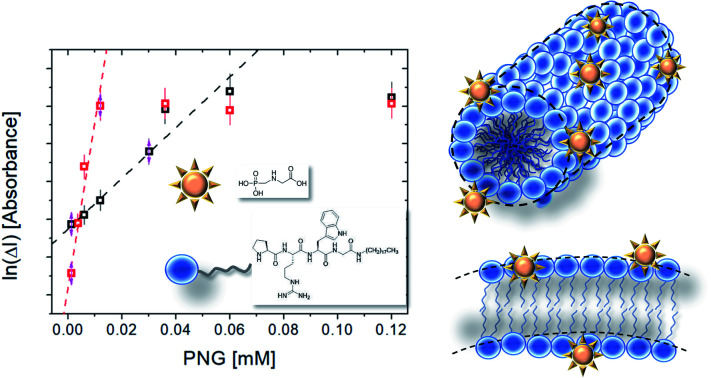

## Introduction

In the last few decades, the use of pesticides in agronomy has increased with the need to control agricultural pests, ensuring increased farm production.^1–3^ However, this widespread use has negatively impacted human health, ecological imbalance, and environmental contamination.^4^ A million people are estimated to be poisoned by pesticides worldwide, of which over 300 000 die each year.^4,5^

The most common type of pesticide used worldwide is organophosphate pesticides (OPs). Among them, glyphosate, *N*-(phosphonomethyl) glycine (PNG), is a compound widely used for weed control in agricultural production and urban, industrial, and recreational areas worldwide.^6–8^ It inhibits the biosynthesis of aromatic amino acids (phenylalanine, tyrosine, and tryptophan), leading to various metabolic disorders, including the disruption of protein production, creation of by-products, and a general metabolic disruption of the phenylpropanoid pathway.^9,10^

OP toxicity in humans is related to its role inhibiting the enzyme acetylcholinesterase (AChE) that promotes the accumulation of the neurotransmitter acetylcholine (AcTh) in the nerves affecting the normal functioning of organs and muscle activities, causing severe symptoms that are potentially deadly.^11^ The action of catalytic peptide nanostructures has been studied to design an artificial enzyme capable of emulation of the catalytic triad of AChE, composed of glutamic acid, which recognizes the choline site group (Ch) as well as histidine, a proton donor, and serine, responsible for pesticide hydrolysis.^12^

Detection of PNG residues is a crucial approach to contamination management. Many methodologies have been used in the literature, such as HPLC,^13^ capillary electrophoreses,^14^ mass spectrometry,^15^ fluorescence spectroscopy,^16^ cyclic voltammetry,^17^ amperometry,^18^ and coulometry.^19–22^ Among these, colorimetric sensors are interesting because they have easy operation, visual quantification, and low sample volume.^20,23^ Recently, many groups have developed colorimetric response materials with high sensitivity for PNG detection and high specificity.^19–22^

The most convenient and sophisticated technological tools able to perform fast, simple, low-cost, specific, and highly sensitive detection are biosensors.^24,25^ These analytical devices incorporate a biological component capable of specifically recognizing targets and producing a measurable signal related to the analyte concentration.^26^ One solution explored is the construction of biomimetic sensors, whose biological recognition element is an artificial or synthetic biomolecule that mimics the molecular functionality of interest.^27–29^

**Fig. 1:**
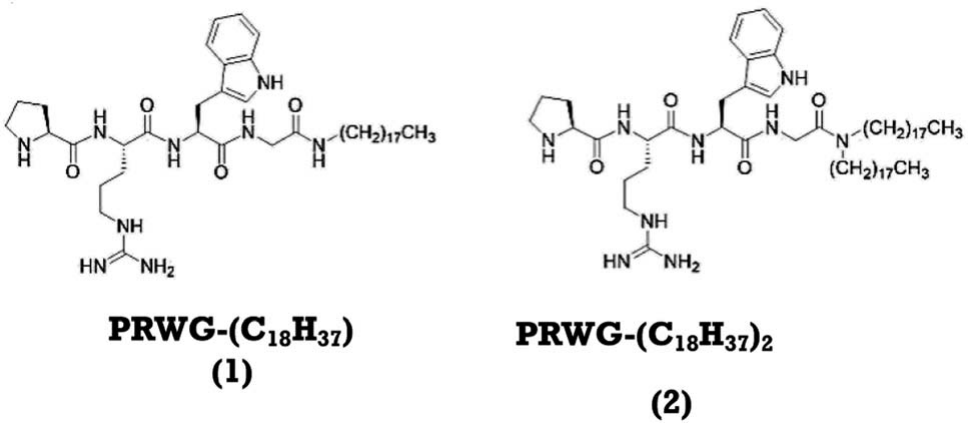
Molecular structures of the lipopeptides PRWG–C_18_H_37_ (compound 1), PRWG–(C_18_H_37_)_2_ (compound 2).

Here we propose a new lipopeptide sequence to mimic the function and structural aspects of AchE. We have studied the interaction between lipopeptides with PNG pesticides and their potential use for analytical applications. These lipopeptides contain the amino acids l-proline (P), l-arginine (R), l-tryptophan (W), and l-glycine (G), covalently linked to one,^30,31^ or two long aliphatic chains,^30–32^ referred to herein as compound 1 and 2, respectively (Fig. 1).

To this end, small-angle X-ray scattering (SAXS) was used to probe structural aspects and provide insights into the mechanisms involved in the lipopeptide self-assembly. Combined with calorimetry (isothermal titration calorimetry, ITC), spectroscopy (circular dichroism, CD) and fluorescence, and microscopy (transmission electron microscopy, TEM and cryo-TEM), we investigate the physicochemical mechanisms involved in the self-assembly of lipopeptide nanostructures and their interaction with PNG. We showed an efficient mimetic behaviour of these lipopeptides as a model of AChE activity, confirmed by Ellman analysis, a well-known method for verifying AChE pesticide interaction.^33^ Also, we observed the influence of the hydrophobicity of compounds 1 and 2 in the PNG limit of detection and sensitivity from a calibration experiment.

## Materials and methods

### Lipopeptides

The lipopeptides used in this study were custom synthesized by Peptide Protein Research Ltd. (Fareham, UK) with purity > 95%, assessed by High-Performance Liquid Chromatography (HPLC). The theoretical and experimental molecular weight (*M*_w_), determined by mass spectrometry, is shown in Table 1. All reagents were purchased from Sigma-Aldrich and used without further purification.

**Table 1:** The lipopeptides used in this study with the theoretical and experimental molecular weight

Compounds	Structural abbreviation	Theoretical *M*_w_	Experimental *M*_w_
1	PRWG–((CH)_2_)_17_CH_3_	766.57	766.65
2	PRWG–[((CH)_2_)_17_CH_3_]_2_	1018.85	1018.86

### Fluorescence spectroscopy

Fluorescence spectra were recorded as described previously.^34^ Pyrene assays were performed using 3.25 × 10^−4^ to 0.13 wt% peptide in 9 × 10^−5^ wt% pyrene solution.

### Circular dichroism (CD)

CD spectra were recorded as described previously.^35^ Quartz cuvettes (thickness of 0.1 mm) were used for the experiments. Ellipticity is reported as the mean residue ellipticity ([*θ*], in degrees cm^2^ dmol^−1^) and calculated as [*θ*] = *M*_rw_ × [*θ*]_obs_/(10 × *c* × *l*), where [*θ*]_obs_ is the ellipticity measured in millidegrees, *M*_rw_ is the mean residue molecular weight of the peptide (molecular weight divided by the number of amino acid residues), *c* is the concentration of the sample in mg mL^−1^, and *l* is the optical path length in centimetres.

### Cryogenic transmission electron microscopy (cryo-TEM)

Cryo-TEM images were obtained as described previously.^34^

### Small-angle X-ray scattering (SAXS)

SAXS experiments were performed on the bioSAXS B21 beamline at Diamond Light Source, UK, and BM29 beamline at ESRF (Grenoble, France), using previously described protocols.^36^

### Isothermal titration calorimetry (ITC)

The calorimetric measurements were performed using a MicroCal iTC200 instrument (Malvern, UK). The sample cell was filled with 200 μL of buffer solution or 0.3 mM of lipopeptide, and the titration syringe contained approximately 40 μL of 2 mM PNG solution. Each experiment consisted of 19 injections with a volume 2 μL each into the sample cell at 25 °C. The measured heat flux signal was integrated over time using Origin™ software to obtain the molar enthalpy values of the enthalpogram. Because the PNG and buffer enthalpogram (the “background”) was found not to be a straight line, it was not directly subtracted from the PNG and lipopeptide enthalpograms. Therefore, the data presented herein is the measured data.

### UV-vis spectroscopy

An Ellman's test was performed to determine the efficiency of the lipopeptide as a mimic of AChE. It is based on the reaction between 5,5′-dithiobis(2-nitrobenzoic acid) (DTNB) and thiocholine (TCh) to form a yellow anion (5-thio-2-nitrobenzoate) (TNB^2−^) during enzymatic reactions at *λ* = 412 nm (*ε* = 13 600 mol^−1^ L cm^−1^). All the experiments were carried out using a Cary 7000 UV-vis-NIR spectrometer (Agilent) connected to a rapid mixing stopped-flow accessory. We verified that all reactions happened in the first 2 minutes. To this end, we prepared two stock solutions, one with the lipopeptide (compound 1 or 2) at 0.06 mM mixed with DNTB at 0.9 mM, both in phosphate buffer, and the other composed of AcTh at 1.8 mM in phosphate buffer. All reactions were carried out in a quartz cuvette with 80 μL of solution. For the kinetic experiments, we fixed the wavelength at 412 nm and observed the absorbance intensity as a function of the time (for around 30 min).

### Thermochemical DFT calculations

Calculations were done with the ORCA program version 4.2.1,^37,38^ employing the B3LYP^39–42^ functional and ma-def2-TZVP basis set^43,44^ (named B3LYP/def2-TZVP/SMD/D3BJ). The SMD system was implicitly used to model the solvent of water and cyclohexanone,^43^ and the Becke–Johnson damping (D3BJ) was used for the dispersion correction.^45,46^ VMD software^47^ was used for the visualization of optimized geometries. We performed Nudged Elastic Band (NEB)^48^ calculations from optimized triple zeta geometries using a slightly larger ma-def2-TZVP basis set. NEB calculations were used to estimate a minimum energy path (MEP) along transition geometries from reactant to products.

## Results and discussion

**Fig. 2:**
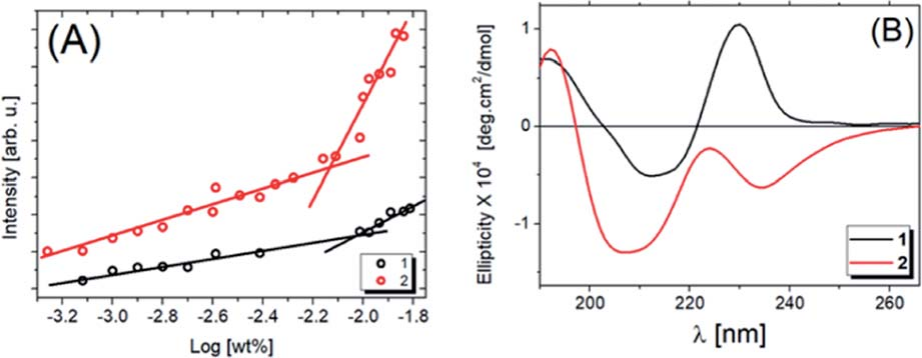
Fluorescence intensity results using pyrene as probe (378 nm) for compounds 1 and 2 (A) as a function of the concentration. The intersection point of the two straight lines determines the cac values. (B) CD spectra for 0.033 wt% 1 and 2 in water solutions at the pH 7.

Fluorescence experiments were carried out to determine the critical aggregation concentration (cac) of compounds 1 and 2, as shown in Fig. 2A. The fluorescence intensity of pyrene as a fluorescence probe is plotted as a function of the logarithm of the peptide concentration evaluated at 378 nm from the fluorescence spectrum (Fig. S1A and B†). The intersection of two lines (Fig. 2A) determines the cac. For compound 1 the cac value is 9.33 × 10^−3^ wt%, and for compound 2, it is 7.41 × 10^−3^ wt%, the latter being the more hydrophobic as it has two alkyl chains. Comparing the cac values obtained previously at pH 3.5,^31^ with those reported in this study at pH 7.0, we noted that the solubility increases with increasing pH.

**Fig. 3:**
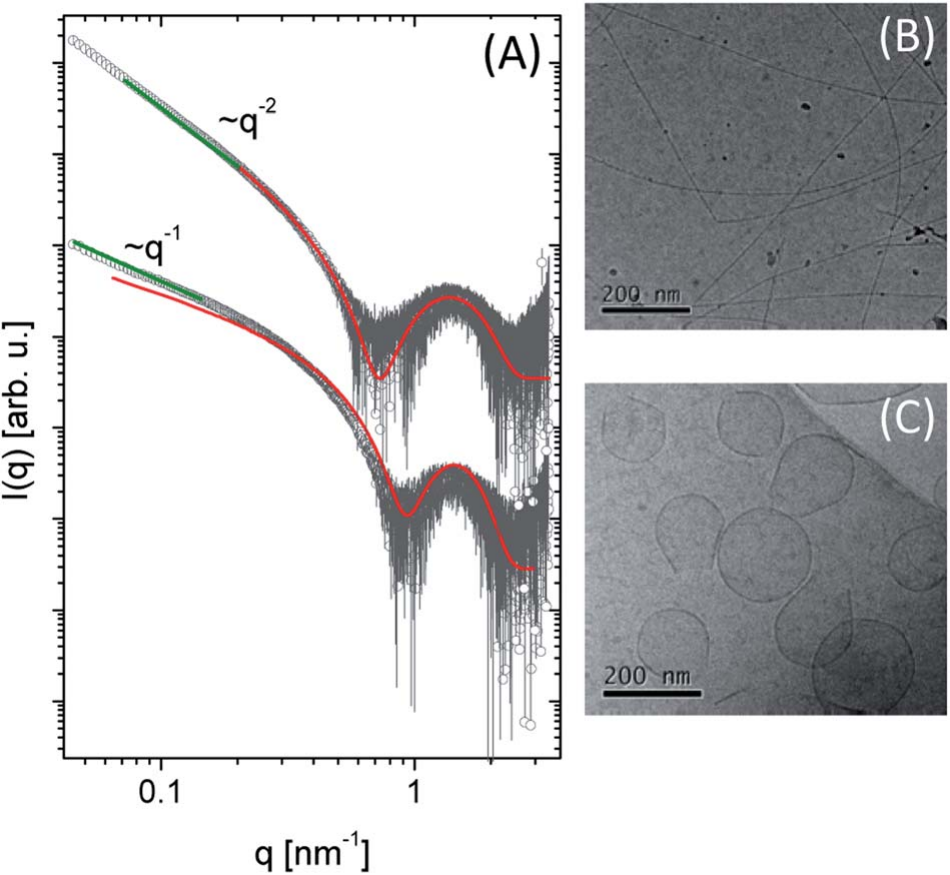
(A) SAXS data spectra of 1 and 2 at 0.2 wt%. The green line represents the power law slope at low *q* values, corresponding to cylinder (*q*^−1^) and planar (*q*^−2^) assemblies. The red line is the fitting using a cylinder core–shell or bilayer as form factor. Cryo-TEM images were obtained for (B) compound 1 and (C) compound 2 at pH 7.0.

The secondary structures were analyzed by CD experiments, as shown in Fig. 2B. For compound 1, a maximum peak around 190 nm is observed and associated with π–π* stacking interactions related to the formation of weak β-sheets, while the minimum peak at ∼210 nm is also due to β-sheet structure, although the position of this band is shifted. The maximum at ∼230 nm is due to the presence of tryptophan.^49,50^ For compound 2, we observe the same band around 190 nm and an overall change in the ellipticity profile. Likely, the presence of one extra carbon chain changes the molecular packing and, consequently, the chirality of the system, which promotes modifications in the CD signal. As evidence of this hypothesis, the minimum at ∼205 nm followed by a maximum band at ∼223 nm is close to the behaviour expected for proline, as previously observed.^51^ In this case, the red-shift of the peaks in the spectrum for 2 (Fig. 2B) may be due to aggregation or self-assembly.^52^ Comparing these results with the ones already published,^53^ it is noted that the increase of the solution pH promotes a considerable modification in the secondary structure of compound 2. Also, the number of alkyl chains influences the lipopeptide secondary structure. To obtain more information about this, SAXS experiments were performed, the data being shown in Fig. 3A (open circles).

The scattering intensity (*I*(*q*)) for both molecules presents a form factor maximum at a wavenumber *q* = 1.5 nm^−1^, characteristic of core–shell structures. However, it is possible to observe different intensity behaviour in the low *q* region: for 1 the intensity decays as *q*^−1^ (green line), suggesting a cylindrical structure.^54^ In the case of compound 2, a planar structure (*q*^−2^ power law decay) is indicated. More quantitative information about the systems was obtained by fitting the experimental data using the SASView program using a cylinder core–shell or a bilayer form factor, respectively (Fig. 3A). The fit parameters are summarized in Table 2. For 1, we obtained a cylinder diameter (2*δ*_T_ + 2*δ*_H_) around 6.46 nm and for 2 the thickness of the bilayer is 5.3 nm (Fig. S2†). Comparing the head and the tail length between them, we observed the values obtained for 2 are smaller than 1 showing this structure is more compact than 1. At pH = 3.5, compound 1 self-assembles in spherical micelles.^31^ Here, at pH 7, cylindrical micelles were observed, while compound 2 forms bilayers at both pH values.

**Table 2:** The SAXS parameters were obtained by the data fitting using the cylinder and bilayer model. *δ*_T_ and *δ*_H_ are the thickness of the hydrophobic and hydrophilic region, respectively, whereas Δ*ρ* is the scattering length contrast of the hydrophilic region relative to the hydrophobic one, *i.e.*, Δ*ρ* = *δ*_H_/*δ*_T_

	Compound 1 (pH 7.0)	Compound 2 (pH 7.0)	Compound 1 (pH 3.5)^a^	Compound 2 (pH 3.5)^a^
*δ*_T_ [nm]	2.26 ± 0.21	1.81 ± 0.18	1.44	2.2
*δ*_H_ [nm]	0.97 ± 0.09	0.84 ± 0.13	1.2	2.0
Δ*ρ*	−1.72 ± 0.16	−1.63 ± 0.56		

a Results extracted from ref. 31.

The morphology of these lipopeptides in 0.5 wt% solutions was assessed by cryo-TEM (Fig. 3B and C). For compound 1, elongated structures 9.2 nm in diameter were observed (Fig. 3B), comparable with the dimensions obtained by SAXS form factor fitting. On the other hand, for compound 2, planar vesicle-like structures with an average diameter of around 170 nm were observed (Fig. 3C). The presence of bilayer structures is consistent with the SAXS data analysis. Interestingly, irregular planar structures were observed at acidic pH,^31^ while compound 2 in a neutral medium presents vesicle-like structure.

The catalytic activity of the lipopeptides was determined by spectrophotometric experiments using Ellman's test.^28,29,33^ The acetylthiocholine chloride (AcTh) concentration range was 2 × 10^−4^ to 6 × 10^−3^ mmol L^−1^, and the lipopeptide concentration was 0.04 mmol L^−1^ in pH 7.0 phosphate buffer solution. The formation of the anion (5-thio-2-nitrobenzoate) (TNB^2−^) was qualitatively observed through the yellowish color of the mixture (Fig. S3A†) and confirmed by UV/vis spectroscopy increasing the amount of AcTh led to an increase in absorbance at 412 nm (Fig. S3B†).

To explain the role of micelles in promoting AcTh decomposition, we hypothesize that the N terminus of proline acts as a nucleophile. In contrast, TFA, present in all lipopeptide salts, could serve as a proton acceptor in a general base catalysis mechanism. We note that this is not the only possible mechanism for the change in the proline protonation state. The micelle environment can also alter the protonation equilibrium due to the reduced water content and the presence of many positively charged groups. However, this second hypothesis was not explored in this work.

Molecular dynamics results^30^ indicate at least three distinct environments in approximately spherical micellar lipopeptide systems: the apolar core, an intermediate hydrophilic interface region, and the bulk. These regions have different chemical environments and can influence the l-proline protonation equilibrium. Previously,^31^ we have computed the proton transfer equilibrium between TFA and a proline amide model molecule in three distinct environments: water, cyclohexanone, and *n*-heptane modeling the outside, the amino acid–aqueous interface, and the micelle core, respectively.

**Fig. 4:**
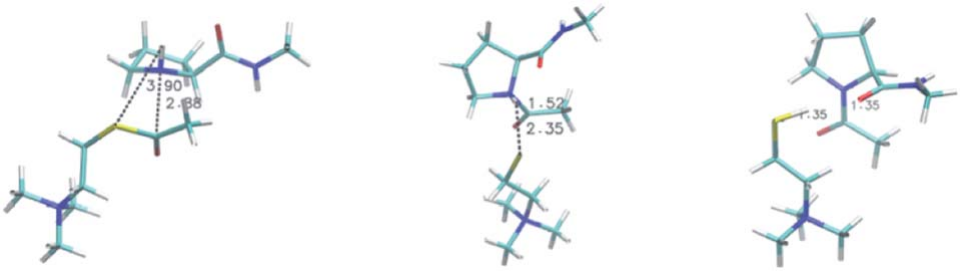
Reactant complex (left), transition state (center) and product (right) for the *N*-methyl-l-prolinamide and acetylthiocholine reaction. Geometries were obtained using the B3LYP/def2-TZVP/SMD/D3BJ methodology as detailed in the text. Distances (shown in figure) for the nascent C–N and S–H bonds are; reactant: C–N 3.90 Å, S–H 2.88 Å; transition state C–N 1.52 Å, S–H 2.35 Å; product C–N 1.35 Å, S–H 1.35 Å.

To investigate our mechanistic hypothesis, we compute reaction free energies for the AcTh tetrahedral intermediate formation, following nucleophilic attack by proline and AcTh hydrolysis reaction. In our investigation, the peptide is modeled as a single *N*-methyl-l-prolinamide (Pro-NMe) molecule, a methyl-capped proline amino acid. The tetrahedral intermediate formed after the Pro-NMe attack is 10.9 kcal mol^−1^ higher in energy (reaction (2), Table 3) when compared to the reactant complex (shown in Fig. 4). On the other hand, the complete hydrolysis reaction is exergonic, with Δ*G* values of −2.27 and −4.24 kcal mol^−1^ in water and cyclohexanone. Both reactions are weakly affected by the medium. However, the l-proline proton equilibrium with TFA is highly medium dependent, Δ*G* changing from a positive 14.4 kcal mol^−1^ to 4.35 kcal mol^−1^ when going from an aqueous environment (bulk) to less polar cyclohexanone (micelle interface region).

**Table 3:** Reaction-free energies computed using DFT and the B3LYP/def2-TZVP/SMD/D3BJ methodology described in the text. Pro(H) stands for (protonated) *N*-methyl-l-prolinamide, TFA(H) stands for (protonated) trifluoroacetic acid, Pro–AcTh for the tetrahedral intermediate, ThC for thiocholine, and Ac for protonated acetic acid. Energies in kcal mol^−1^

Reaction		Water	Cyclohexanone
1	ProH + TFA → Pro + TFAH	14.4	4.3
2	Pro + AcTh → Pro–AcTh	10.9	10.6
3	AcTh + H_2_O → ThC + AcH	−2.3	−4.2

This result suggests that proline-containing lipopeptides could act as nucleophiles due to the shift in protonation equilibrium enabled by the micellar environment, similar to that present on the AChE enzyme. We used DFT and NEB to investigate the thiocholine formation step, where acyl transfer occurs after the attack by proline. This mechanism has a reaction barrier of 12.8 kcal mol^−1^ in cyclohexanone (model of the micelle interface region), with the key steps being the acyl transfer followed by proton transfer from the nitrogen of proline to the leaving thiocholine. The high-energy zwitterionic character of the transition state (a NEB climbing image is shown in Fig. 4) is quickly stabilized by the proton transfer. The reaction energy profile generated from our NEB calculations is shown in Fig. S4.† We also investigated a general base catalysis mechanism, a single-step reaction where TFA actively takes part but found much more significant barriers. As expected, the corresponding enzymatic acylation mechanism features a lower 7.2 kcal mol^−1^ barrier computed using QMMM.^55^

**Fig. 5:**
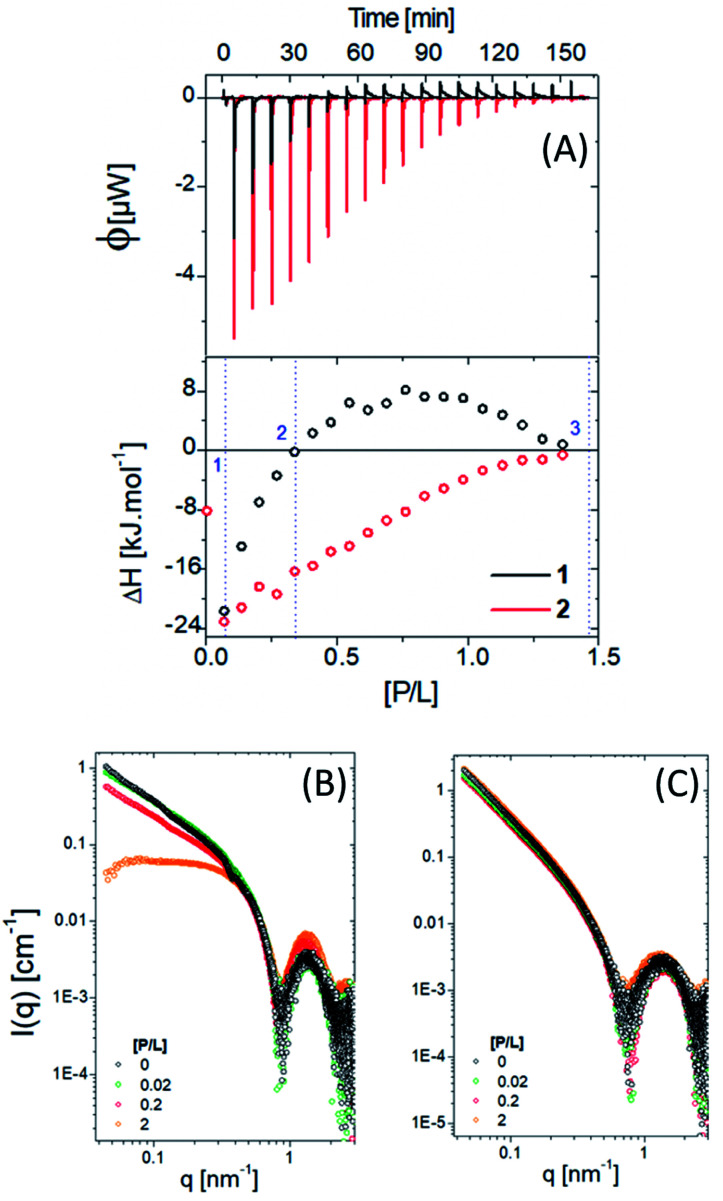
(A) ITC data for molecules 1 and 2 in the presence of PNG and the correspondent SAXS data (B) and (C), respectively.

Fig. 5A shows ITC results for titration of PNG into 1 and 2 at pH 7. The obtained enthalpogram (bottom plot) for each system as a function of molar ratio pesticide/lipopeptide (P/L), obtained from the integration of each peak shown in the fluxogram (upper plot), is different, which points to distinct interactions. Titration of PNG with compound 1 (black open circles) is exothermic (Δ*H* < 0) in all investigated ranges of P/L. Moreover, there is a practically linear dependence of Δ*H* with P/L, the interaction being less exothermic the higher the P/L value. On the other hand, the mixture of PNG with compound 2 (red open circles) is only exothermic for P/L < 0.3, being endothermic otherwise. In contrast to the case with 1, the dependence of Δ*H* with P/L is nonlinear, with a maximum of Δ*H* at approximately P/L = 0.7. Interestingly, in both cases, Δ*H* ∼ 0 (meaning no interaction) is reached at the same P/L ≈ 1.3. It is known that exothermic processes are, in general, related to electrostatic interactions, while endothermic ones have a hydrophobic component.^56^ Compound 2 has a more hydrophobic environment than sample 1. To confirm the specificity between glyphosate and the two lipopeptides studied herein, we performed an ITC assay using the pesticide carbetamide in the same experimental conditions used before (Fig. S5†). We observed Δ*H* ∼ 0 for all P/L ratios for both lipopeptides, showing a weak interaction between carbetamide and 1 and 2 and thus indicating high specificity between PNG and the lipopeptides.

SAXS experiments were carried out to determine how the presence of the pesticide modifies the lipopeptide self-assembly (Fig. 5B and C). Three different P/L ratios were chosen: (1) P/L = 0.02 (blue line), corresponding to the first ITC injection; (2) P/L ∼ 0.2, where Δ*H* ∼ 0 and (3) P/L = 2, associated with the reaction saturation.

For 1, increasing the amount of pesticide, the SAXS data changes mainly in the low *q* region due to a morphological transition from cylinders/fibrils to spheres (with a characteristic plateau in the intensity at low *q*), which may explain the nonlinear behaviour of the obtained ITC enthalpogram (Fig. 5B, black circles). On the other hand, no morphological changes were observed for compound 2, corroborating the predictable and stable behaviour of the obtained enthalpogram (Fig. 5B, red circles).

UV/vis kinetic experiments were performed to quantify the lipopeptide catalytic activity. Fig. S6A and B† present the absorbance curves *vs.* time, at 412 nm, for several concentrations of the respective peptides. Two different behaviours are observed: an exponential increase followed by a flat region after a few seconds. The slope of the curve in the first region represents the reaction rate of the formation of thiocholine (TCh), while the flat part indicates the reaction saturation. The slope significantly increases for compound 1 compared to 2. After 0.15 min, the value is constant for all reactions, but the absorbance increases with the lipopeptide concentration.

**Fig. 6:**
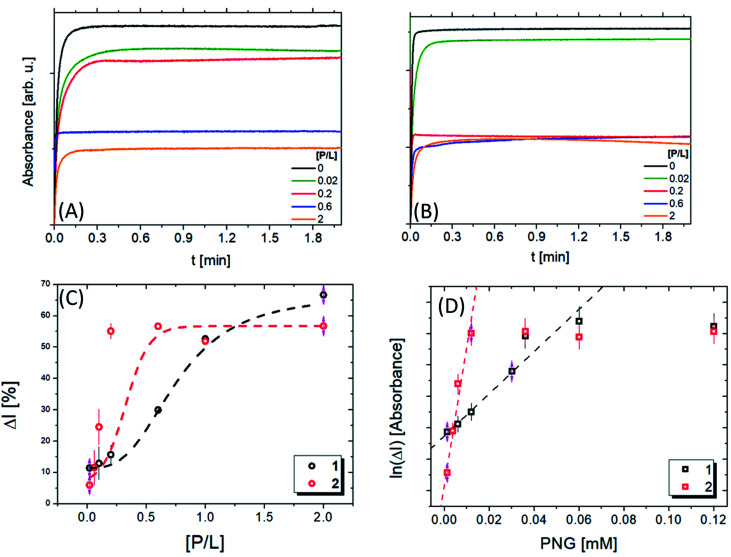
Absorbance intensity as a function of the time at different [P/L] ratios for compounds 1 (A) and 2 (B), respectively. (C) Inhibition ratio (Δ*I*) as a function of [P/L] for compounds 1 (A) and 2, the red and black lines are fitted using a sigmoidal function to obtain the IC_50_ values. (D) The ln(Δ*I*) as a function of the PNG molar concentration for compounds 1 (A) and 2, the red and black lines are linear fits to determine the limit of detection (LOD).

Fig. 6A and B present the kinetic experiments in the presence of pesticides. The slope decreases with increasing [P/L], corroborating the interaction between lipopeptide and the pesticide. Therefore, the pesticide associated with the peptide sequence decreases AcTh conversion. These changes are more noticeable in the system containing 1. We calculated the inhibition ratio (Δ*I* = *A*_[P/L]_/*A* × 100) by the variation of the value as a function of [P/L] (Fig. 6C). A linear behaviour was observed around 1.2–30 μmol L^−1^, which is compatible with other materials described in the literature^19–23^ (Table 4). Interestingly, for 1 a value Δ*I* around (62 ± 2.5)% was obtained. For 2, up to P/L = 0.2 Δ*I* reaches a maximum value of (57 ± 1.5)%, showing 1 is more susceptible than 2 in the presence of PNG.

**Table 4:** Comparison of present method with other platform sensors using UV-vis methods for PNG detection

Compound	Linear region (mM)	LOD (mM)	Ref.
Cd–PVA^a^	0.1–1.2	0.6	19
MNBZ–Ag NPs^b^	0.1–1.2	0.02	20
3,3′,5,5′-Tetramethylbenzidine	2–150	1	21
SiNP/OPD/Cu^2+^^c^	2.5–250	11.8	22
Cysteamine–Au NPs	0.5–7	0.05	23
1	1.2–30	0.3	This work
2	1.2–12	1.5	This work

a Cd–PVA: copper doped poly(vinyl)alcohol.

b MNBZ–Ag NPs: 2-mercapto-5-nitrobenzimidazole capped silver nanoparticles.

c SiNP: silicon nanoparticles with *o*-phenylenediamine (OPD).

Nevertheless, the value of [P/L] required to reach these inhibition ratios is different in each case: for compound 1, it is necessary that [P/L] > 1.5, while for compound 2, [P/L] > 0.5. This result is in agreement with ITC data (Fig. 5B): at [P/L] = 0.0, 0.6 and 2.0, where Δ*I*_1_ ≈ Δ*I*_2_, we also have |Δ*H*_1_| ≈ |Δ*H*_2_|, where | | indicates the absolute value, since Δ*H* can be positive or negative depending on the predominant nature of the interaction (hydrophobic or electrostatic, respectively), as previously mentioned. Note that the maximum value of [P/L] shown in the ITC enthalpogram is ∼1.4, Δ*H* ≈ 0 for higher [P/L] values. At [P/L] ∼ 0.2, Δ*I*_1_ < Δ*I*_2_ in the same way that |Δ*H*_1_| < |Δ*H*_2_|. In conclusion, the inhibition rate is closely linked to the variation in enthalpy of the interaction between PNG and the lipopeptides. The IC_50_ values obtained for the lipopeptides were 46 and 23 μM, for 1 and 2, respectively.

Comparing the IC_50_ values for AChE in the presence of PNG^57,58^ with values found here, our values are 10^3^ less than the enzyme. Therefore, we concluded that lipopeptides are highly sensitive to the pesticide. This may be because one micelle contains around 160 PRWG^30^ lipopeptide molecules, which promotes more interactions between the recognition sites and the pesticide. The limit of detection (LOD) obtained was 0.3 mM and 1.5 mM for 1 and 2 (Table 4), respectively, showing that 1 detects PNG at a concentration 5× less than 2 (Fig. 6D). Comparing these results with LOD values obtained for other colorimetric sensors listed in Table 4, we can see a good agreement among them. The LOD values obtained for compounds 1 and 2 are in the PNG concentration range that is allowed by governmental health institutions.^59^

## Conclusions

We have developed a new peptide sequence capable of mimicking the behaviour of the acetylcholinesterase (AChE) enzyme and with great potential for PNG detection. We observed that the critical aggregation concentration (cac) of compound 2 is lower than 1, indicating that the system is more hydrophobic. This would be expected since molecule 2, which contains two alkyl chains, is more hydrophobic. In addition, significant differences were observed in the CD spectra, showing that changing the packing of the molecules promotes a considerable change in the secondary structure of the hydrophilic peptide sequence. The SAXS data and the cryo-TEM images provide information about the morphology, for 1 cylindrical micelles and vesicle-like structures for 2. Fitting the SAXS data, it was observed that system 2 is slightly more compact than 1, corroborating the fluorescence assays. The Ellman's test results showed that both biomolecules could be used as AChE enzyme mimetic systems. The calculations of computed reaction-free energies indicate that proline can act as a nucleophile due to the shift in protonation equilibrium enabled by the micellar environment in a way similar to that present on the protein. The isothermal titration calorimetry experiments showed that the entropy variation and the interactions between the lipopeptides and the pesticide differ depending on the PNG concentrations. In the presence of the pesticide, a morphological change from a cylindrical to a spherical micellar system was observed for 1, which could explain the large change in the behaviour of the enthalpogram when compared to the lipopeptide with two aliphatic tails.

The kinetic data showed that both systems undergo inhibition processes, and LOD values compatible with other values in the literature^19–23^ were determined (Table 4). An interesting result is that the hydrophobicity of the system influences the LOD value. In the case of the more hydrophobic and compact structure formed by 2, a decrease in the sensor's sensitivity was observed, demonstrating that the supramolecular arrangement is of paramount importance in the pesticide detection process. The PNG in the solution can form a stable complex in the presence of polar organic groups such as phenolic, hydroxylic, carboxylic, and amino functional groups *via* H-bond formation. It was noted that this pesticide can interact with primary and secondary amines present in the peptides.^60^ CD experiments suggest that the amino terminus of proline may undergo a conformation change so that it is less available at the hydrophilic interface, promoting a decrease in the interaction with PNG and thus reducing the concentration of PNG that can be detected.

## Author contributions

BBG: conceptualization, methodology, formal analysis, investigation, writing – original draft, writing – review & editing. PLOF: ITC methodology, formal analysis, investigation, writing – original draft. BC: formal analysis, investigation, writing – original draft. PTS: theory, writing – original draft. MDCN: theoretical and formal analysis, writing – original draft. JS: cryo-TEM experiments. I. W. H: supervision, funding acquisition, writing – review and editing. WAA: conceptualization, supervision, funding acquisition, writing – review and editing.

## Conflicts of interest

There are no conflicts to declare.

## Supplementary Material

NA-004-D2NA00345G-s001
