# Supplementary material for: Interaction between glyphosate pesticide and amphiphilic peptides for colorimetric analysis
Source: Nanoscale Adv. 2022 Jul 28;4(17):3592–9. doi: 10.1039/d2na00345g (PMC9400510; doi:10.1039/d2na00345g)
Supplement: NA-004-D2NA00345G-s001 [file NA-004-D2NA00345G-s001.pdf]

Electronic Supplementary Material (ESI) for Nanoscale Advances.
This journal is © The Royal Society of Chemistry 2022

Support information

Interaction between glyphosate pesticide and amphiphilic peptides for colorimetric analysis

Barbara B. Gerbelli,[a,b] Pedro L. O. Filho,[c,d] Bruna Cortez,[b] Pedro T. Sodré,[b] Mauricio D. Coutinho-Neto,[b] Ian W. Hamley,[a]* Jani Seitsonen,[e] Wendel A. Alves[b]*

[a]University of Reading, Department of Chemistry, Reading, United Kingdom.

[b]Universidade Federal do ABC, Centro de Ciências Naturais e Humanas, São Paulo, SP, Brazil.

[c]University of Copenhagen, Niels Bohr Institute, Copenhagen, Denmark.

[d]Universidade de São Paulo, Instituto de Física, São Paulo, SP, Brazil.

[e]Nanomicroscopy Center , Aalto University, Puumiehenkuja 2, FIN-02150 Espoo, Finland

**Corresponding authors: i.w.hamley@reading.ac.uk; wendel.alves@ufabc.edu.br*

Contents

Figure S1. Raw fluoresce data were performed using 3.25×10^{-4} to 0.13 wt % peptide in 9×10^{-5} wt % pyrene solution to compounds (A) for **1** and (B) for **2**. 2
Figure S2. Cylinder and bilayer SAXS models were used to analyze the data, where δT and δH are the thickness of the hydrophobic and hydrophilic region, respectively, whereas $\Delta\rho$ is the scattering length contrast of the hydrophilic region relative to the hydrophobic one, i.e.,$\Delta\rho = \delta H \delta T$. 2
Figure S3. (a) Picture of the Elman's test for **2** in different AcTh concentrations. (b) The absorbance is a function of the wavelength for different AcTh amounts.........................2
Figure S4.: NEB-CI energy profile with 11 images of AcTh breakage into an acetyl proline and thiocholine (Th). Geometries of reactants (left) and product (right) are shown as image inserts. The NEB trajectory was previously optimized using def2-SVP from ma-def2-TZVP stationary points. The NEB approximation to the transition state has an energy of 12.85 kcal/mol. 3
Figure S5. ITC data for molecules **1** and **2** in the presence of PNG and the Carbetamide (Carb)..3
Figure S6. The kinetic behavior of the absorbance intensity as a function of the time with different lipopeptide amounts of (A) **1** and (B) **2**, respectively. 4

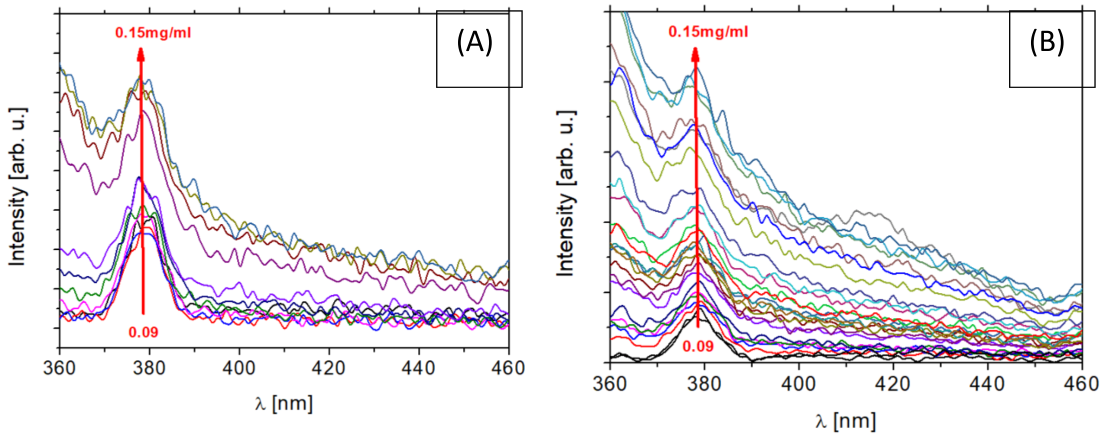

Figure S1. Raw fluoresce data were performed using 3.25 × 10^{-4} to 0.13 wt % peptide in 9 × 10^{-5} wt % pyrene solution to compounds (A) for **1** and (B) for **2**.

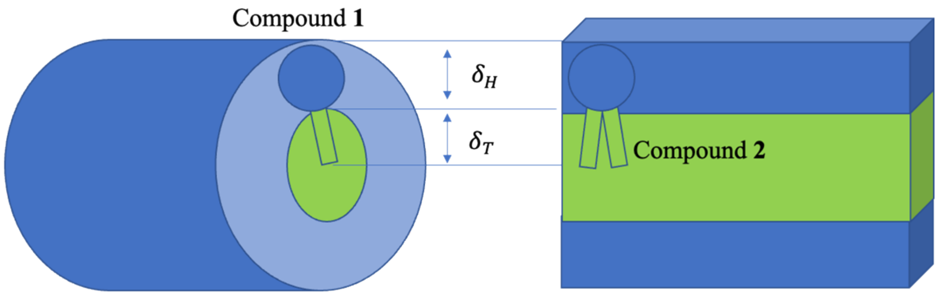

Figure S2. Cylinder and bilayer SAXS models were used to analyze the data, where δ_T and δ_H are the thickness of the hydrophobic and hydrophilic region, respectively, whereas $\Delta\rho$ is the scattering length contrast of the hydrophilic region relative to the hydrophobic one, i.e., $\Delta\rho = \dfrac{\delta_H}{\delta_T}$.

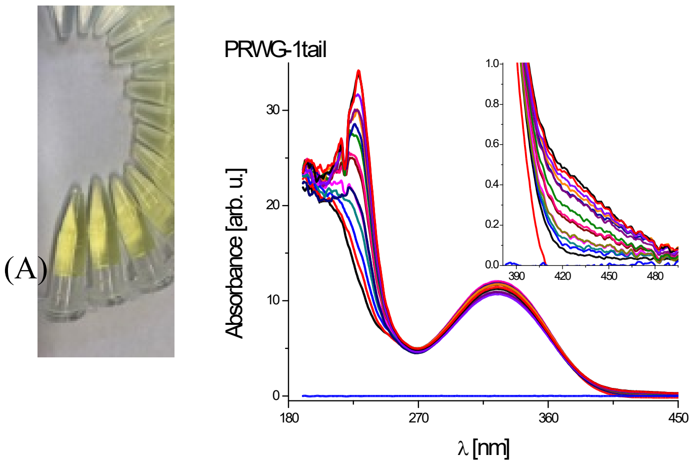

(B)

Figure S3. (A) Picture of the Elman’s test for **2** in different AcTh concentrations. (B) The absorbance is a function of the wavelength for different AcTh amounts.

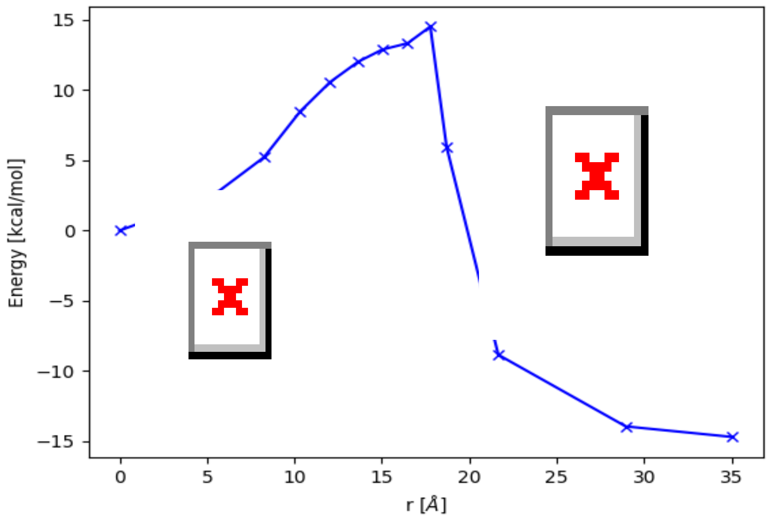

Figure S4.: NEB-CI energy profile with 11 images of AcTh breakage into an acetyl proline and thiocholine (Th). Geometries of reactants (left) and product (right) are shown as image inserts. The NEB trajectory was previously optimized using def2-SVP from ma-def2-TZVP stationary points. The NEB approximation to the transition state has an energy of 12.85 kcal/mol.

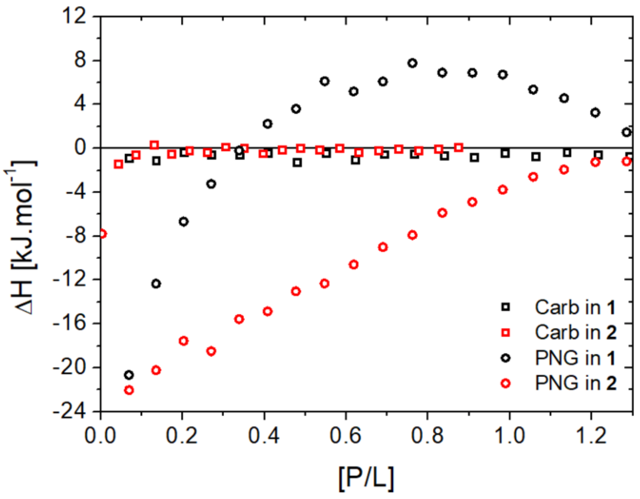

Figure S5. ITC data for molecules **1** and **2** in the presence of PNG and the Carbetamide (Carb).

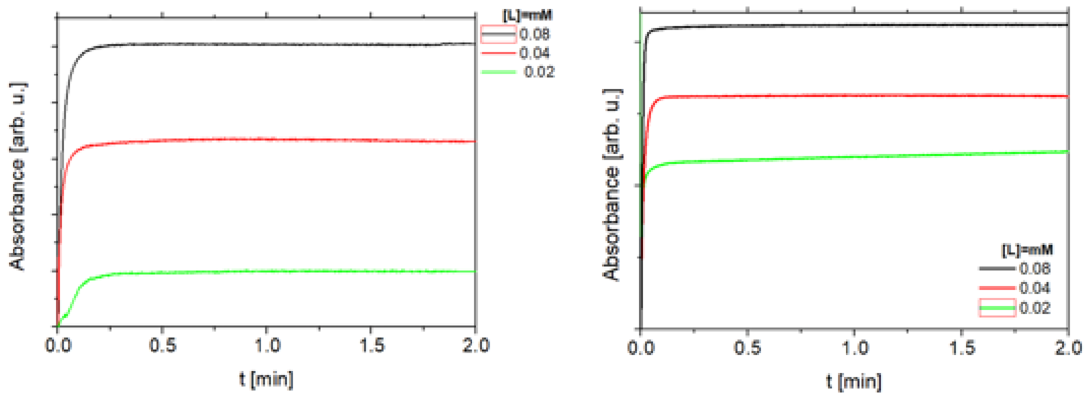

(A) **(B)**

Figure S6. The kinetic behavior of the absorbance intensity as a function of the time with different lipopeptide amounts of (A) **1** and (B) **2**, respectively.
